# PLAN-psoriasis: protocol for a randomised controlled feasibility trial comparing patient-led ‘as-needed’ treatment and therapeutic drug monitoring-guided treatment to continuous treatment for adults with clear or almost clear skin on risankizumab monotherapy for psoriasis

**DOI:** 10.1136/bmjopen-2025-106635

**Published:** 2025-10-10

**Authors:** Weiyu Ye, Kingsley Powell, Niamh Dooley, Charlotte M Thomas, Bola Coker, Helen McAteer, Jessica Ruoheng Wei, Wei Ren Tan, David Baudry, Tejus Dasandi, Jade Pizzato, Tracey H Sach, John Gregory, Zijing Yang, Andrew E Pink, Richard T Woolf, Richard B Warren, John Weinman, Jonathan N Barker, Sarah Chapman, Joseph F Standing, Sam Norton, Catherine H Smith, Satveer K Mahil

**Affiliations:** 1 St John’s Institute of Dermatology, Guy’s and St Thomas’ NHS Foundation Trust & King’s College London, London, UK; 2 Social, Genetic and Developmental Psychiatry Centre, King’s College London, London, UK; 3 Research and Development, Guy's and St Thomas’ NHS Foundation Trust, London, UK; 4 Psoriasis Association, Northampton, UK; 5 School of Primary Care, Population Sciences and Medical Education, University of Southampton, Southampton, UK; 6 Centre for Rheumatic Diseases, King’s College London, London, UK; 7 Dermatology Centre, Northern Care Alliance NHS Foundation Trust & Division of Musculoskeletal and Dermatological Sciences, Manchester Academic Health Science Centre, University of Manchester, Manchester, UK; 8 Centre for Adherence Research and Education, King’s College London, London, UK; 9 Infection, Immunity and Inflammation Research & Teaching Department, UCL Great Ormond Street Institute of Child Health, London, UK

**Keywords:** Clinical Protocols, DERMATOLOGY, Psoriasis, Drug Therapy

## Abstract

**Introduction:**

Targeted biologic therapies have transformed outcomes for individuals with psoriasis, a common immune-mediated inflammatory skin disease. The widespread use of these highly effective treatments has led to a growing number of individuals with clear or nearly clear skin remaining on continuous, long-term treatment. Personalised strategies to minimise drug exposure may sustain long-term disease control while reducing treatment burden, associated risks and healthcare costs. This study aims to evaluate the feasibility of a definitive pragmatic effectiveness trial of two personalised dose minimisation strategies compared with continuous treatment (standard care) in adults with well-controlled psoriasis receiving the exemplar biologic risankizumab.

**Methods and analysis:**

This is a multicentre, assessor-blind, parallel group, open-label randomised controlled feasibility trial in the UK, evaluating two personalised biologic dose minimisation strategies for psoriasis. 90 adults with both physician-assessed and patient-assessed clear or nearly clear skin on risankizumab monotherapy for ≥12 months will be randomised in a 1:1:1 ratio to (1) patient-led ‘as-needed’ treatment, where risankizumab is administered at the first sign of self-assessed psoriasis recurrence, (2) therapeutic drug monitoring-guided treatment, with personalised dosing intervals determined using a pharmacokinetic model or (3) continuous treatment as per standard care, for 12 months. Participants will be invited to submit self-reported outcomes and self-taken photographs every 3 months using a bespoke remote monitoring system (mySkin app) and will attend an in-person assessment at 12 months. They may also request additional patient-initiated follow-up appointments during the trial if needed. The primary outcome is the practicality and acceptability of the two personalised biologic dose minimisation strategies, assessed as a composite measure including recruitment and retention rates, adherence to the assigned strategies and acceptability to both patients and clinicians. The feasibility of collecting healthcare cost and resource utilisation data will also be evaluated to inform a future cost-effectiveness analysis. A nested qualitative study, involving semistructured interviews with patients and clinicians, will explore perspectives on the personalised biologic dose minimisation strategies. These findings will inform the design of a future definitive trial.

**Ethics and dissemination:**

This study received ethical approval from the Seasonal Research Ethics Committee (reference 24/LO/0089). Results will be disseminated through scientific conferences, peer-reviewed publications and patient/public engagement events. Lay summaries and infographics will be codeveloped with patient partners to ensure the findings are accessible for the wider public.

**Trial registration number:**

ISRCTN17922845.

**STRENGTHS AND LIMITATIONS OF THIS STUDY**

- The study will provide data on the feasibility of personalised biologic dose minimisation strategies in well-controlled psoriasis.
- Participants will be recruited from multiple dermatology departments in the UK, including both academic and non-academic centres.
- This study leverages novel digital platforms to streamline trial processes and reduce the need for in-person visits.
- The design and delivery of the study has been informed by extensive patient and public involvement.
- The study is not powered to evaluate clinical effectiveness.

## Introduction

### Background and rationale

 Chronic plaque psoriasis is a common immune-mediated inflammatory disease affecting over 125 million people worldwide.^1^ It is characterised by red, scaly skin plaques and is associated with multiple comorbidities including depression, cardiometabolic syndrome and psoriatic arthritis.^2^ Although there is currently no cure, advances in the understanding of psoriasis pathophysiology have led to the development of biologic therapies targeting key inflammatory pathways involved in disease progression, including tumour necrosis factor (TNF), interleukin-17 (IL-17) and interleukin-23 (IL-23).^2^ Biologic therapies have transformed outcomes for individuals with psoriasis, with up to 60% achieving complete skin clearance at 12 months.^3^,^5^

The widespread adoption of biologic therapies in routine care has resulted in a growing population of individuals with well-controlled psoriasis receiving continuous, long-term treatment. Although highly effective, biologic therapies are associated with dose-dependent adverse events, including increased susceptibility to infections and, more rarely, serious complications such as demyelination.^6^,^9^ These risks remain a concern for patients: during the COVID-19 pandemic, a global cross-sectional survey found that one in four patients discontinued biologic treatment, with nearly half citing concerns over long-term immune suppression.^10^ The high cost of biologic treatment and the burden associated with ongoing injections and hospital visits further highlight the need for optimised treatment strategies that reduce drug exposure while sustaining disease control. Although recent surveys indicate support among dermatologists for biologic dose minimisation in patients with well-controlled psoriasis, real-world implementation remains variable due to limited robust, high-quality evidence to guide safe and effective dose reduction without increasing the risk of disease flares.^11 12^

Emerging evidence indicates that durable disease control may be achievable through strategies that reduce biologic exposure. Several studies have shown that disease control can often be maintained for extended periods following biologic discontinuation and is typically regained quickly on retreatment, without major safety concerns.^413^,^17^ A recent systematic review reported median times to relapse ranging from 12 to 34 weeks following biologic discontinuation, with longer periods of disease control observed after cessation of IL-23 inhibitors.^18^ Notably, in most studies, biologic discontinuation occurred relatively early in the treatment course (typically between 12 and 28 weeks), shortly after disease control was achieved.^1315^,^17^ This raises the possibility that maintaining disease control for a longer duration prior to discontinuation may lead to even more favourable outcomes.

To date, most biologic dose minimisation studies in psoriasis have employed standardised, stepwise dose reduction protocols, applying the same tapering schedule across all participants.^19 20^ While structured, this ‘one-size-fits-all’ approach does not account for individual variation. As healthcare models seek to become increasingly personalised, there is a need for more flexible, individualised strategies. One such approach involves adjusting the biologic dosing interval based on individual response, so further doses are only given if disease control is no longer maintained.^21^ As self-reported skin status is a reliable method for monitoring psoriasis,^22^ patients are well positioned to detect disease recurrence themselves through the appearance of a skin plaque. This opens the possibility of a patient-led ‘as-needed’ approach, in which biologic treatment is paused and restarted by the patient at the first sign of recurrence.^23^ This strategy may enhance patient empowerment and reduce treatment burden; however, its safety and effectiveness in real-world clinical settings remain untested.

Another personalised approach involves using serum drug levels to guide dosing. Pharmacokinetic (PK) data across multiple classes of biologics including IL-23, IL-17 and TNF inhibitors indicate that in individuals who are established on treatment, serum drug concentrations often exceed the levels required for disease control.^24^,^26^ An exposure–response analysis of the exemplar IL-23 inhibitor biologic risankizumab in phase II and phase III trials found that average serum drug concentration, rather than trough level, was the stronger predictor of treatment response.^26^ Patients with higher average serum risankizumab concentrations were more likely to achieve disease control, suggesting that serum drug level-guided dose adjustments may allow individualised dose reductions without compromising efficacy.^26^ We have developed a model-informed precision dosing approach using Bayesian estimation to predict individual PK parameters to generate personalised dosing intervals for risankizumab (via a user-friendly online dashboard).^27^ However, the acceptability of implementing therapeutic drug monitoring (TDM)-guided biologic dosing in routine psoriasis care, and the feasibility of delivering a definitive pragmatic effectiveness trial, remains to be established.

### Objectives

The primary objective of this study is to determine the practicality and acceptability, to both patients and clinicians, of two personalised biologic dose minimisation strategies compared with continuous treatment in adults with chronic plaque psoriasis who have been clear or nearly clear for ≥12 months on risankizumab monotherapy. The treatment strategies are:

- Patient-led ‘as-needed’ treatment: risankizumab is self-administered at the first self-assessed sign of psoriasis recurrence, supporting patient empowerment and self-management.^23^
- TDM-guided treatment: a PK model-informed precision dosing approach is used to generate a personalised dosing interval (via an online dashboard), based on serum risankizumab concentration and individual covariates.^27^
- Continuous treatment: ongoing fixed interval risankizumab dosing, as per standard care.

The secondary objectives are to:

- Provide preliminary data on the potential clinical effectiveness, safety, tolerability and treatment burden of (1) patient-led ‘as-needed’ treatment and (2) TDM-guided treatment compared with (3) standard care (continuous treatment). These data will allow estimation of effect sizes to inform sample size calculations for a future definitive trial.
- Assess the feasibility of collecting data on healthcare costs and resource use for each treatment strategy.
- Refine the model-informed precision dosing dashboard for TDM-guided risankizumab dosing using longitudinal PK data and pharmacodynamic (PD) data, to enhance its future utility.^27^

The exploratory objectives are to:

- Determine the feasibility of self-sampling blood, which may be used in future research and clinical care.
- Determine the feasibility of genotype testing, which may be used in future research and clinical care.

## Methods and analysis

### Study design and setting

**Figure 1:**
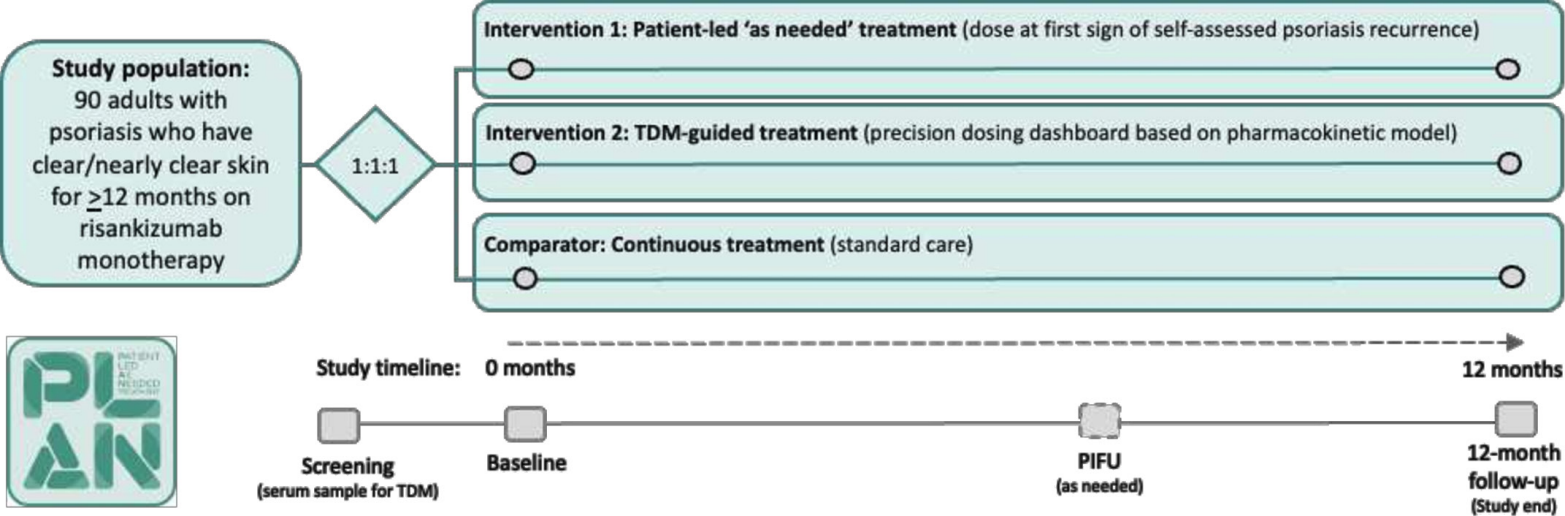
Trial design. PIFU, patient-initiated follow-up; TDM, therapeutic drug monitoring.

Patient Led As Needed (PLAN)-psoriasis is a multicentre, assessor-blind, parallel group, open-label, feasibility randomised controlled trial (RCT) with economic scoping and a nested qualitative study (figure 1). It will be conducted across multiple hospital dermatology departments in the UK, representing a mix of academic and non-academic centres (online supplemental table 1).

### Participants and recruitment

**Figure 2:**
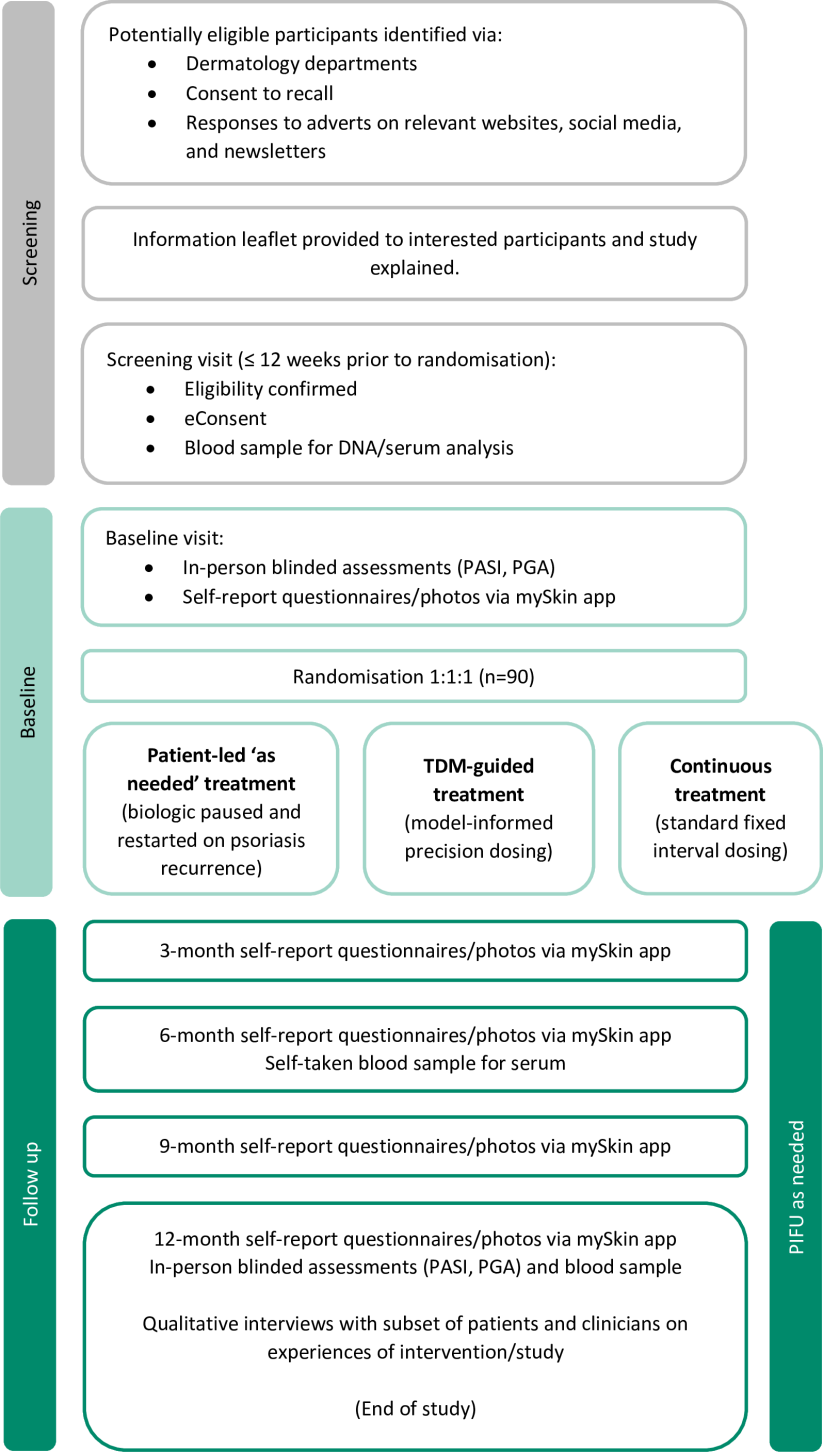
Participant flow chart. PASI, Psoriasis Area and Severity Index; PGA, Physician Global Assessment; PIFU, patient-initiated follow-up; TDM, therapeutic drug monitoring.

Participants are eligible for the trial if they meet the inclusion and exclusion criteria outlined in table 1. Figure 2 summarises the flow of participants through the study.

**Table 1:** Eligibility criteria

Inclusion criteria	Exclusion criteria
Adults aged ≥16 years with a diagnosis of chronic plaque psoriasis with evidence of clear or nearly clear skin on risankizumab monotherapy for ≥12 months. Physician Global Assessment of clear or nearly clear skin at study entry. Clinician assessed Psoriasis Area and Severity Index ≤2 at study entry. Patient Global Assessment of clear or nearly clear skin at study entry.	Receiving risankizumab for a primary indication other than psoriasis. Concomitant immune-modifying therapy or phototherapy.

Potentially eligible participants will be identified by the clinical team at each study site during routine clinical care visits, as well as from individuals scheduled to attend the sites. Additionally, relevant research databases will be searched, and individuals with psoriasis who have consented to be contacted about related research studies will be approached. To maximise reach, study details will also be disseminated through national and regional networks, including the UK Dermatology Clinical Trials Network, the Psoriasis Association, relevant websites, newsletters and social media channels. In line with the National Institute for Health and Care Research’s (NIHR’s) Research Inclusion Strategy, efforts will be made to engage with individuals from diverse backgrounds to ensure the study population is representative of the wider psoriasis population.^28^ ^29^ To further promote inclusivity, individuals will also have an opportunity to self-refer into the study.

All individuals who express interest will have the opportunity to discuss the study with a member of the research team and will be provided with an information sheet. They will be given sufficient time to consider their participation. Written informed consent will be obtained electronically (eConsent) from each participant prior to study enrolment by a member of the research team. Table 2 outlines the study procedures in the trial.

**Table 2:** Study procedures for Patient Led As Needed (PLAN)-psoriasis

	Screening	Baseline		Month 3	Month 6	Month 9	Month 12	PIFU
		Baseline and randomisation	Training					
Informed consent	X							
Eligibility	X	X						
Demographics	X							
Medical history	X	X	X	X	X	X	X	X
Blood sample*	X				†		X	X
Randomisation		X						
Education‡			X					
Clinical assessments (in-person)								
PASI	X	§					§	X
Physician Global Assessment	X	§					§	X
Body mass index		X						
Waist circumference		X						
Patient reported outcome measures								
Patient Global Assessment	X	X		X	X	X	X	X
DLQI			X	X	X	X	X	X
EQ-5D-5L			X	X	X	X	X	X
Itch NRS			X	X	X	X	X	X
PHQ			X	X	X	X	X	X
GAD			X	X	X	X	X	X
BIPQ			X	X	X	X	X	X
PsAID			X	X	X	X	X	X
PEST			X	X	X	X	X	X
Injection log			X	X	X	X	X	X
Photographs			X	X	X	X	X	X
Adverse events recording				X	X	X	X	X
Adherence and acceptability questionnaires					X		X	
Resource use			X	X	X	X	X	X
Nested qualitative study								
Semistructured interviews¶							X	

* Serum albumin, creatinine, C reactive protein, risankizumab concentration, anti-risankizumab antibody titre.

† Self-taken blood sample.

‡ Training provided on treatment strategy, PIFU and the mySkin app.

§ Assessor-blind skin assessments.

¶ Subset of participants and clinicians only.

BIPQ, Brief Illness Perception Questionnaire; DLQI, Dermatology Life Quality Index; EQ-5D-5L, EuroQol 5-Dimension 5-Level; GAD, Generalised Anxiety Disorder Questionnaire; NRS, Numerical Rating Scale; PASI, Psoriasis Area and Severity Index; PEST, Psoriasis Epidemiology Screening Tool; PHQ, Patient Health Questionnaire; PIFU, Patient-Initiated Follow-Up; PsAID, Psoriatic Arthritis Impact of Disease.

### Randomisation and blinding

Participants will be randomised (1:1:1 ratio) to one of three treatment strategies: (1) patient-led ‘as-needed’ treatment, (2) TDM-guided treatment and (3) continuous treatment (standard care). Randomisation will be conducted via a secure online system to ensure allocation concealment. Stratification will be based on recruiting site and clear or nearly clear skin status, with randomly varying block sizes used to maintain allocation concealment and achieve balanced group sizes across these categories.

Although participants and the research team will not be blinded to the treatment strategy allocated, disease severity assessments, including the Psoriasis Area and Severity Index (PASI) and Physician Global Assessment, will be conducted by an assessor who is blinded to treatment allocation and is not involved in the participant’s care. Participants will be asked not to reveal their allocated treatment strategy to this blinded assessor. The blinded assessor will be either a dermatologist or a dermatology nurse.

### Interventions

Following randomisation, all participants will receive training on their assigned treatment strategy, which they will follow for the 12-month study period. Topical treatments can be used as usual across all treatment strategies. Routine follow-up is scheduled at month 12 (study end), after which participants will return to National Health Service (NHS) care. Participants may continue with their assigned treatment strategy beyond the study period if they wish, following discussion with their clinical team.

Participants will be invited to use the mySkin app to submit self-reported outcomes and self-taken photographs at three-monthly intervals throughout the 12-month study period. Disease severity at each timepoint will be assessed using the self-reported Patient Global Assessment. This approach is supported by prior research demonstrating concordance between patient and clinician-assessed disease severity in psoriasis, especially at low disease activity.^30^ No routine in-person follow-up visits are scheduled prior to the final follow-up at month 12.

The mySkin app is designed to streamline trial procedures, promote patient empowerment and minimise unnecessary clinic visits. It enables remote collection of patient-reported outcomes and self-taken photographs (including step-by-step guidance to optimise/standardise photograph capture). The app also includes an injection log to record administered biologic injections and displays each participant’s assigned treatment strategy, along with a personalised dashboard showing when their next biologic dose is due.

The mySkin app provides step-by-step instructions for self-sampling of blood for serum analysis (aligned with a supporting video, https://bit.ly/taking-blood-sample). All participants will be invited to collect up to 1 mL of blood using serum microtubes at month 6. Self-sampling kits will be mailed to participants’ homes in advance, and they will be asked to return their samples to the central study team using prepaid packaging. Participants will also be invited to complete an in-app questionnaire to assess the acceptability of self-sampling of blood, incorporating relevant constructs from the theoretical framework of acceptability.^31^

Throughout the study, participants may request ad hoc patient-initiated follow-up (PIFU) visits via the mySkin app if they rate their psoriasis severity as moderate or worse based on the Patient Global Assessment, or if they have concerns about any intercurrent issues. PIFU visits will occur within 5 working days of participant request.

For participants unable to use the mySkin app, the research team will arrange alternative methods of data collection such as paper forms. Participants without access to a smartphone will be provided with an electronic device and the necessary training. All participants may also access further support from the research team via telephone if needed.

#### Intervention arm 1: patient-led ‘as-needed’ treatment

Participants randomised to patient-led ‘as-needed’ treatment will pause risankizumab monotherapy and resume it at the first sign of self-assessed psoriasis recurrence to maintain clear or nearly clear skin, keeping a minimum dosing interval of 12 weeks^23^. Recurrence is defined as Patient Global Assessment Score of mild (ie, no longer clear/nearly clear).

#### Intervention arm 2: TDM-guided treatment

For participants randomised to TDM-guided treatment, a clinician will use an online precision dosing dashboard incorporating a PK model for risankizumab therapy to calculate a personalised dosing interval, which will be applied throughout the 12-month study period.^27^ To generate this interval, the dashboard requires the serum concentration of risankizumab and the anti-risankizumab antibody titre, measured using routine rapid chemiluminescence assays following the screening visit. The dashboard also requires the date of the last risankizumab dose, body weight, PASI and the serum levels of albumin, creatinine and C reactive protein.

#### Comparator arm: continuous treatment (standard care)

Participants randomised to the comparator arm will continue risankizumab monotherapy at fixed 12-weekly intervals, as per standard care.

### Outcomes

#### Primary outcome

The primary outcome will be the practicality and acceptability of the two personalised biologic dose minimisation strategies, assessed as a composite measure comprising recruitment and retention rates, as well as adherence to and acceptability of the allocated treatment strategy (table 3).^31^

**Table 3:** Components of the primary outcome

Component	Evaluation criteria
Recruitment rate	Proportion of eligible individuals invited to participate who are randomised.
Retention rate	Proportion of participants who complete the 12-month follow-up visit.
Adherence	Adherence to the allocated treatment strategy, assessed using patient and clinician questionnaires.
Acceptability	Acceptability of the allocated treatment strategy, using relevant constructs from the theoretical framework of acceptability.^31^ This will be assessed through patient and clinician questionnaires, with a subset of participants also taking part in one-to-one semistructured qualitative interviews.

These components will inform whether progression to a full-scale, definitive RCT is appropriate:

- If all components exceed 80%, the study will proceed to a definitive RCT without protocol modifications.
- If any component falls between 20% and 80%, the study may still progress, but with protocol modifications.
- If any component is below 20%, progression to a full trial will be halted.

These progression criteria were developed through study team consensus and may be refined during the study with input from patients, clinicians and researchers, under the guidance of the trial steering committee (TSC).

#### Secondary outcomes

The secondary outcomes include:

- Clinical and healthcare outcomes over the 12-month period, comparing the different groups based on the following:
  - Number of weeks per patient spent with ‘disease control’. Disease control is defined as a Patient Global Assessment Score of clear or nearly clear skin with no evidence of disease worsening.
  - Number of ‘disease worsening’ episodes per patient. Disease worsening is defined as an assessor blind increase in PASI of ≥3 from baseline with a minimum PASI of 5 (assessed via in-person visits), and/or a treatment change such as biologic dose escalation, biologic switch or the addition of adjunctive therapy. This definition has been previously used in RCTs of immune-mediated inflammatory diseases, including psoriasis.^32^
  - Disease severity at the end of the trial, assessed through blinded PASI and Physician Global Assessment at the month 12 in-person visit.
  - Patient-reported outcomes including the following:
    - Dermatology Life Quality Index.
    - EuroQol 5-Dimension 5-Level Questionnaire (EQ-5D-5L).
    - Itch Numerical Rating Scale.
    - Patient Health Questionnaire for depression.
    - Generalised Anxiety Disorder questionnaire.
    - Brief Illness Perception Questionnaire.
    - Psoriatic Arthritis Impact of Disease Questionnaire, if participants have a rheumatologist confirmed diagnosis of psoriatic arthritis.
  - Treatment and healthcare burden, assessed by total drug exposure, number of drug-free weeks per patient (post each 12-week cycle) and number of PIFU visits.
  - Safety and tolerability, assessed by:
    - Incidence of adverse events and serious infections.
    - Incidence of new onset psoriatic arthritis (screened for using the Psoriasis Epidemiology Screening Tool) or flare of psoriatic arthritis (as confirmed by a rheumatologist).
    - Proportion of patients with antidrug antibodies.
    - Number of injection site reactions.
- Feasibility of collecting healthcare cost and resource use data for each treatment strategy assessed by the proportion of missing data.
- Acceptability and practicality of follow-up completion, measured by:
  - Proportion of participants who complete the three-monthly self-assessments (including submission of photographs) using the mySkin app.
  - Proportion of missing data per participant.
  - Duration of in-person study visits at month 12 and during PIFU visits.
- Refine the model-informed precision dosing dashboard for TDM-guided biologic treatment using longitudinal PK and PD data, to enhance its future utility.^27^
- Acquisition of clinical samples and data for future use by the wider scientific community.
- Acceptability and practicality of self-taken blood samples for serum analysis, assessed by questionnaires evaluating relevant constructs from the theoretical framework of acceptability, proportion of participants who submit a self-taken blood sample following study team invitation, time from invitation to sample receipt by study team, proportion of self-taken samples from which the serum drug concentration is successfully measured and qualitative interviews with a subset of participants.^31^
- Acceptability and practicality of genotype testing, assessed by participant questionnaires evaluating relevant constructs from the theoretical framework of acceptability and qualitative interviews with a subset of participants.^31^

#### Adverse events

Adverse event data will be collected during in-person study visits and through self-reported questionnaires embedded within the mySkin app. Expected events of special interest will be captured using follow-up self-report questionnaires, including (1) worsening of psoriasis and (2) worsening or emergence of psoriatic arthritis. These events will be reported as trial outcomes rather than adverse events.

### Sample size

As this is a feasibility trial, a formal sample size calculation is not appropriate. On a pragmatic basis, a total of 90 participants (30 participants per treatment arm) has been deemed sufficient to estimate each component of the primary outcome, including recruitment rate, retention and acceptability and adherence in the intervention arms. Specifically, the overall retention rate has an estimated 95% CI of approximately ±10%, and acceptability and adherence within each treatment arm will have an estimated interval of approximately ±20%.

Recruitment will be monitored closely to ensure adequate participant enrolment to meet the target sample size. In the event of recruitment challenges, the TSC will advise on appropriate strategies to improve recruitment, for example, by revising the eligibility criteria or implementing targeted recruitment campaigns.

### Statistical methods

All analyses will follow the intention-to-treat principle. As this is a feasibility trial, the study is not powered to detect differences in outcomes between groups. The primary focus of the analysis will be on estimation to inform the design of a future pragmatic effectiveness trial. Appropriate descriptive statistics will be used to summarise quantitative data, both overall and by treatment arm (eg, means and SD for continuous variables, frequencies and percentages for categorical variables). At each postrandomisation timepoint, each clinical outcome for each treatment group will be summarised using suitable descriptive statistics. No subgroup analyses are planned.

To explore the potential effects of the allocated treatment on clinical outcomes, linear mixed-effects regression models will be used. Each model will include a random intercept to account for repeated measures within participants, with fixed effects for treatment group, time (as a categorical variable) and their interaction to estimate between-group differences at each timepoint. Adjusted mean differences between groups will be reported, along with 80% and 95% CIs.

Loss to follow-up will be reported by treatment arm and assessment timepoint. Missing data will be summarised using descriptive statistics. The primary analysis will use all available data without imputation, under the assumption that data is missing at random. The extent and pattern of missingness will be examined to inform the design of a future trial.

Sensitivity analyses, including per-protocol analyses, may be conducted where appropriate to explore the impact of adherence and protocol deviations. In accordance with the Consolidated Standards of Reporting Trials (CONSORT) extension for pilot and feasibility studies, no formal hypothesis testing will be undertaken and p values will not be reported.^33^

### Economic scoping

To inform the design of a full economic evaluation comparing different personalised biologic dose minimisation strategies, this feasibility trial will focus on developing and piloting data collection methods to facilitate this. Specifically, it will:

- Pilot the collection of data needed to estimate intervention costs, with feasibility assessed through completion rates of individual items and overall.
- Develop and implement a self-report resource use questionnaire within the mySkin app to capture (1) broader NHS resource use related to psoriasis and (2) personal costs, including the impact on a participant’s ability to work. Data will be collected at baseline and every 3 months, with completion rates for individual items and the overall questionnaire used to evaluate feasibility.
- Assess the feasibility of using the EQ-5D-5L embedded in the mySkin app to estimate utility values. As all five domains need to be completed to assess utility, the completion rates for each domain will be examined.

Resource use will be valued using published unit cost data for the most recent price year available. This will enable preliminary identification of potential cost drivers across the different treatment strategies, helping to prioritise key data collection areas in the future definitive trial. As this is a feasibility study, no incremental cost-effectiveness analysis will be conducted.

### Nested qualitative study

#### Aims

The nested qualitative study aims to support interpretation of quantitative findings by exploring patient and clinician perspectives on the acceptability and real-world practicality of patient-led ‘as-needed’ and TDM-guided biologic dose minimisation strategies. In addition, it seeks to generate new insights into the barriers and enablers influencing adherence to these approaches, as well as factors affecting trial recruitment and retention.

#### Methods

At least 16 participants will be recruited for one-to-one semistructured interviews, with a minimum of 8 from each intervention arm. Participants will be purposively sampled to ensure diversity in demographic and clinical characteristics, as well as variation in the number of biologic injections received during the study. Efforts will also be made to include participants who withdrew from the trial or who encountered challenges with the interventions or research processes. From the pool of trial participants who consented to be contacted about the qualitative study, individuals meeting the sampling criteria will be provided with an information leaflet. Those who agree to take part will be asked to complete a separate consent form for the interview.

At least eight clinicians from participating sites will also be recruited for one-to-one semistructured interviews, including doctors, nurses and pharmacists. Each clinician will receive an information leaflet, and if willing to participate, will be asked to complete a separate interview consent form.

Interviews will be conducted by a trained member of the research team, either face-to-face, by telephone or via video conference call, according to participant preference. A semistructured topic guide will be used to explore relevant constructs from the theoretical framework of acceptability, experiences of psoriasis management and perspectives on trial procedures and interventions, with flexibility to explore emergent themes.^31^ Interviews are expected to last 45–60 min and will be audio recorded (with permission), transcribed verbatim and deidentified to maintain confidentiality.

#### Analysis

Interview transcripts will be analysed using framework analysis, informed by the theoretical framework of acceptability.^31^ Coding will be primarily deductive, organised around the key constructs of the theoretical framework of acceptability, while allowing for inductive insights to capture unanticipated factors influencing intervention acceptability and trial participation. The qualitative findings will be triangulated with the main trial results to inform recommendations for implementing trial findings and help shape the design of a future definitive trial.

### Data collection and management

Data obtained at in-person visits will be recorded on case report forms and entered onto CAPTURE (ChArting PaTient outcomes Using an online REsource), a secure electronic capture and sample management system hosted by Guy’s and St Thomas’ NHS Foundation Trust. This system complies with all relevant regulatory requirements. Site staff at each recruiting site will be responsible for data entry, which will include clinical information and blood sample records. Self-reported data including photographs will be collected through the mySkin app, which is hosted on RADAR-base, an open-source mobile health platform for collecting, monitoring and analysing data from wearables and mobile technologies.^34^

Data collection forms will be programmed with validation rules to minimise data entry errors and enable real-time data checking at sites. In addition, the central research team will regularly review entered data and issue data clarification requests to sites as needed.

All blood samples will be transferred to King’s College London (KCL) for processing and/or storage according to standardised operating procedures. Aliquots of serum and DNA will be stored securely and in accordance with appropriate protocols.

For long-term follow-up and health economic research, study data will be linked to external datasets held by national healthcare providers, including Hospital Episodes Statistics, mortality, Clinical Practice Research Datalink and National Cancer Registry Service. The study will also link data to other research studies that participants have previously contributed to.

### Retention of participants

To promote participant retention, the local team at each site will contact participants to reschedule any missed in-person appointments. Automated email reminders will be sent on days 5 and 10 if patient-reported outcomes have not been completed in the mySkin app at each scheduled 3-month follow-up timepoint throughout the 12-month study period. Participants will also be reimbursed for reasonable travel expenses incurred when attending study visits that fall outside of their routine clinical care.

Participants may withdraw from the study at any time without affecting their medical care. They may choose to (1) withdraw from the intervention only, where they still provide study-specific data at follow-up timepoints or (2) withdraw from the study entirely. Participants will be informed at the time of initial study consent that any clinical information, samples and data generated from those samples collected prior to withdrawal of consent will be retained by the research team and cannot be withdrawn.

### Study oversight

A TSC will provide oversight of the study and ensure that its aims are delivered to time and target. The TSC will include an independent chair, an independent statistician and two patient representatives. As this is a non-Clinical Trial of Investigational Medicinal Product using risankizumab, which has been used in routine care since 2019, the functions of the data monitoring committee will be incorporated into the remit of the TSC. The study is co-sponsored by King’s College London (lead co-sponsor) and Guy’s and St Thomas’ NHS Foundation Trust.

### Patient and public involvement (PPI)

This study aligns with the James Lind Alliance research priorities for psoriasis, including how to induce remission, treat flares and regain disease control.^35^ The design of this feasibility trial has been shaped by extensive PPI, including a dedicated meeting with PPI advisors, a national patient scoping survey publicised by the Psoriasis Association and feedback from a patient panel from the NIHR Research Design Service. The trial will also be promoted via organisations such as the Psoriasis Association, the UK’s leading member organisation for people affected by psoriasis.

We have an established PPI group comprising members from diverse sex, ethnic and socioeconomic backgrounds, in line with the principles of inclusive research.^28^ PPI members have contributed to the design of key study materials, including the patient information sheet, self-sampling guide and the mySkin app. PPI members will continue to be involved throughout the study, including participation in the TSC, reviewing emerging findings and coproducing lay summaries, newsletters and infographics. These materials will be disseminated through patient organisations and social media to maximise reach and ensure the study findings are accessible to the wider public.

## Ethics

Ethical approval for this study has been obtained from the Seasonal Research Ethics Committee (reference 24/LO/0089). In addition, approval was obtained from the local Research and Development office at each site prior to recruitment. Amendments to the protocol will be communicated to the relevant parties as part of regular updates on study progress.

## Dissemination

The study results will be reported and disseminated widely to all stakeholders through presentations at international clinical and scientific conferences, publications in peer-reviewed scientific journals and engagement with the public and patient community via multimedia channels, including the networks and websites of the Psoriasis Association.

## Data storage and sharing

To protect confidentiality, data will be stored in accordance with the Data Protection Act 2019 and the UK General Data Protection Regulation. The study team will retain exclusive access to the data until the publication of major outputs is complete. Deidentified data may be available subject to direct request.

## Discussion

The growing population with clear or nearly clear psoriasis on continuous biologic therapy presents a timely opportunity to re-evaluate current treatment models. There is a need to develop strategies that optimise the use of expensive biologics while reducing unnecessary drug exposure. More efficient use of biologics through personalised dose minimisation strategies could improve access to biologics for a broader group of patients with psoriasis and other immune-mediated inflammatory diseases, while also reducing the risk of adverse events and injection burden for those with stable well-controlled disease.

This feasibility trial will assess the real-world practicality and acceptability of two personalised biologic dosing strategies: (1) patient-led ‘as-needed’ treatment and (2) TDM-guided treatment, compared with standard care. Although risankizumab is the exemplar therapy, the findings are likely to be generalisable to other biologics.

The results of this study, including insights from the nested qualitative study and economic scoping, will inform the design of a future definitive RCT to evaluate the clinical effectiveness, cost-effectiveness and tolerability of biologic dose minimisation strategies. Although this trial is UK-based, the findings are likely to have global relevance, with the potential to reshape long-term psoriasis management, reduce healthcare costs, decrease treatment burden and deliver broader benefits for healthcare systems, patients and society.

The first participant was randomised in December 2024. Recruitment is currently ongoing, with follow-up expected to complete by July 2026. Results are anticipated in early 2027.

## Supplementary material

10.1136/bmjopen-2025-106635 online supplemental table 1
