# Supplementary material for: PLAN-psoriasis: protocol for a randomised controlled feasibility trial comparing patient-led ‘as-needed’ treatment and therapeutic drug monitoring-guided treatment to continuous treatment for adults with clear or almost clear skin on risankizumab monotherapy for psoriasis
Source: BMJ Open. 2025 Oct 10;15(10):e106635. doi: 10.1136/bmjopen-2025-106635 (PMC12519699; doi:10.1136/bmjopen-2025-106635)
Supplement: online supplemental table 1 [file bmjopen-15-10-s001.docx]

**Supplementary Table 1. List of UK participating sites for PLAN-psoriasis.**

| **Number** | **Site** |
| --- | --- |
| 1 | Guy's and St Thomas' NHS Foundation Trust |
| 2 | Epsom and St Helier University Hospitals NHS Trust |
| 3 | Newcastle Upon Tyne Hospitals NHS Foundation Trust |
| 4 | Northern Care Alliance NHS Foundation Trust |
| 5 | Kingston Hospital NHS Foundation Trust |
| 6 | Lewisham and Greenwich NHS Trust |
| 7 | Barts Health NHS Trust |
| 8 | Royal Berkshire NHS Foundation Trust |
| 9 | University Hospitals Bristol and Weston NHS Foundation Trust |
| 10 | The Dudley Group NHS Foundation Trust |
| 11 | East Suffolk and North Essex NHS Foundation Trust |
| 12 | University Hospitals Sussex NHS Foundation Trust |
| 13 | Royal Devon University Healthcare NHS Foundation Trust |
| 14 | Cambridge University Hospitals NHS Foundation Trust |
