# Supplementary material for: PLAN-psoriasis: protocol for a randomised controlled feasibility trial comparing patient-led ‘as-needed’ treatment and therapeutic drug monitoring-guided treatment to continuous treatment for adults with clear or almost clear skin on risankizumab monotherapy for psoriasis
Source: BMJ Open. 2025 Oct 10;15(10):e106635. doi: 10.1136/bmjopen-2025-106635 (PMC12519699; doi:10.1136/bmjopen-2025-106635)
Supplement: Supplementary file 2 [file bmjopen-2025-106635.reviewer_comments.pdf]

PEER REVIEW HISTORY

BMJ Open publishes all reviews undertaken for accepted manuscripts. Reviewers are asked to complete a checklist review form and are provided with free text boxes to elaborate on their assessment. These free text comments are reproduced below.

ARTICLE DETAILS

Title (Provisional)

PLAN-psoriasis: Protocol for a randomised controlled feasibility trial comparing patient-led ‘as needed’ treatment and therapeutic drug monitoring guided treatment to continuous treatment for adults with clear or almost clear skin on risankizumab monotherapy for psoriasis.

Authors

Ye, Weiyu; Powell, Kingsley; Dooley, Niamh; Thomas, Charlotte M; Coker, Bola; McAteer, Helen; Wei, Jessica Ruoheng; TAN, WEI REN; Baudry, David; Dasandi, Tejus; Pizzato, Jade; Sach, Tracey H; Gregory, John; Yang, Zijing; Pink, Andrew E; Woolf, Richard T; Warren, R B; Weinman, John; Barker, Jonathan; Chapman, Sarah; Standing, Joseph F.; Norton, Sam; Smith, C H; K Mahil , Satveer

---

VERSION 1 - REVIEW

**Reviewer**	**1**
**Name**	**Balser, Sigrid**
**Affiliation**	**Formycon AG, Clinical Development & Operations**
**Date**	**02-Sep-2025**
**COI**	**None**

A more detailed review & some suggestions can be found in the attached file.

Overall Assessment bmjopen-2025-106635 (PLAN-psoriasis protocol)

This is a well-designed and timely protocol addressing a clinically important and underexplored area: personalised biologic dose minimisation in psoriasis. The trial is methodologically robust, patient-centred, and includes innovative digital monitoring strategies. The authors clearly articulate the rationale, objectives, and design features. It has the potential to lay the foundation for a definitive trial with major implications for clinical practice, healthcare costs, and patient quality of life.

Major Strengths

1. **Relevance and Novelty**
    - The study tackles a pressing clinical and economic issue: long-term biologic use in patients with clear or nearly clear psoriasis.
    - It is the *first RCT* directly comparing two distinct dose minimisation strategies (patient-led vs. TDM-guided) against continuous treatment.
    - Patient involvement and empowerment are central, which aligns with current

healthcare priorities.

2. **Innovative Methodology**
   - Integration of the **mySkin app** for eConsent, remote monitoring, patient-reported outcomes, and self-sampling represents a forward-thinking approach.
   - The use of a **PK-based precision dosing dashboard** is highly innovative and may serve as a model for broader biologic therapy optimisation.
3. **Comprehensive Design**
   - The trial incorporates quantitative (RCT), qualitative (nested interviews), and economic (cost/resource analysis) components, offering a rich, multidimensional evaluation.
   - Clear **progression criteria** for moving to a definitive RCT (e.g., recruitment, retention, adherence thresholds).
4. **Patient and Public Involvement (PPI)**
   - PPI has been extensive and meaningful, shaping both design and dissemination. This increases the trial's relevance and likely enhances recruitment/retention.

Some Suggestions

1. **Sample Size and Power**
   - While appropriate for feasibility, the sample size (n=90) limits exploration of even exploratory clinical outcomes.
   - Suggestion: Clarify whether any subgroup analyses (e.g., psoriatic arthritis presence, socioeconomic status) will be attempted, even descriptively, to inform the future full-scale trial.
2. **Definition of Outcomes**
   - The **primary outcome** (feasibility composite) is well justified, but thresholds (20– 80%) could be further explained — why these cut-offs? Are they consistent with other feasibility studies in dermatology?
   - Disease worsening is defined partly by PASI increase ≥3 with baseline ≥5. This may exclude subtle but clinically meaningful worsening. Authors could justify this threshold more fully.
3. **Digital Platform Risks**
   - Heavy reliance on the mySkin app for reporting, photographs, and self-sampling could disproportionately affect participants with lower digital literacy.
   - Suggestion: Outline contingency plans for participants unable/unwilling to engage digitally (e.g., telephone support, paper forms).
4. **Therapeutic Drug Monitoring Arm**
   - The dashboard approach is promising, but the protocol lacks detail on how discrepancies between model predictions and clinical judgement will be managed. Will clinicians be able to override dosing recommendations? If so, how will this be recorded and analysed?

5. **Generalisability**
    - While UK-based, the implications are international. However, access to risankizumab and TDM assays may differ elsewhere. Authors could expand discussion on global applicability.

Conclusion

This is a **well-conceived, rigorous, and patient-centred feasibility protocol**. It addresses a highly relevant clinical and economic question, integrates novel digital and pharmacological tools, and is grounded in strong patient and public involvement. With minor clarifications — particularly on outcome thresholds, digital inclusivity, and TDM clinician decision-making — the protocol will provide an excellent foundation for a future definitive trial.

VERSION 1 - AUTHOR RESPONSE

We are very grateful for the constructive comments provided following peer review. We have addressed each point in a detailed, point-by-point response and believe that these revisions have improved the clarity, context and overall quality of the manuscript, ensuring its relevance to the journal's readership.

Comments	Author's Response	Position in marked version
**Reviewer 1**		
**Sample Size and Power**<br>• While appropriate for feasibility, the sample size (n = 90) limits exploration of even exploratory clinical outcomes.<br>• **Suggestion:** Clarify whether any subgroup analyses (e.g., psoriatic arthritis presence, socioeconomic status) will be attempted—even descriptively—to inform the future full-scale trial.	Thank you for your constructive comments. As this is a feasibility study with a total sample size of 90 participants (30 per arm), we have not planned any subgroup analyses, as the numbers will be too small to provide meaningful insight. We have clarified this in the statistical methods section by explicitly stating that no subgroup analyses are planned.	Line 453
**Definition of Outcomes**<br>• The primary outcome (feasibility composite) is well justified, but thresholds (20–80%) could be further explained. Why were these cut-offs chosen? Are they consistent with other feasibility studies in dermatology?	Thank you for your comments. The thresholds for progression to a full-scale, definitive randomised controlled trial were determined by expert consensus and are broadly similar to those used in our recent feasibility trial on the use of Janus Kinase inhibitors in palmoplantar pustulosis (Gleeson D, Chapman S, McAteer H, et al Janus kinase	Line 369

	inhibitors in palmoplantar pustulosis: a mixed-methods feasibility (JAKPPPOT) trial protocol<br>BMJ Open 2025;15:e106361. doi: 10.1136/bmjopen-2025-106361). These thresholds may also be refined during the study with oversight from the Trial Steering Committee. We have revised the manuscript to clarify the rationale for selection of these thresholds.	
**Definition of Outcomes**<br>• Disease worsening is defined partly by PASI increase ≥3 with baseline ≥5. This may exclude subtle but clinically meaningful worsening. Authors could justify this threshold more fully.	Disease worsening is defined as an assessor-blind increase in PASI of ≥3 from baseline with a minimum absolute PASI of ≥5, and/or a treatment change such as biologic escalation, biologic swich or the addition of adjunctive therapy. This definition was selected because it has been used previously in a multicentre randomised controlled trial of immune-mediated inflammatory diseases, including psoriasis, and reflects a balance between capturing clinically meaningful disease worsening and avoiding misclassification due to minor or transient fluctuations in PASI scores. This rationale is outlined in the manuscript and we have also provided the relevant reference (31).	Lines 380-385
**Digital Platform Risks**<br>• Heavy reliance on the mySkin app for reporting, photographs, and self-sampling could disproportionately affect participants with lower digital literacy.<br>• **Suggestion:** Outline contingency plans for participants unable or unwilling to engage digitally (e.g., telephone support, paper forms).	Thank you, we agree that contingency plans are important for participants who are are unable or unwilling to engage digitally. In such cases, telephone support will be available through the research team, who can also arrange alternative methods of data capture such as paper forms. For participants without a smartphone, the research team	Lines 328-331

	can provide a suitable electronic device along with the necessary training. We have revised the manuscript to clarify these contingency plans.	
**Therapeutic Drug Monitoring Arm**<br>• The dashboard approach is promising, but the protocol lacks detail on how discrepancies between model predictions and clinical judgement will be managed. Will clinicians be able to override dosing recommendations? If so, how will this be recorded and analysed?	All participants randomised to the therapeutic drug monitoring guided treatment arm will be advised to take risankizumab according to the personalised dosing intervals recommended by the dashboard. Treating clinicians will not be able to override these dosing recommendations. As adherence is a key aspect of feasibility, participants' adherence to the personalised dosing intervals will be monitored throughout the trial.	N/A
**Generalisability**<br>• While UK-based, the implications are international. However, access to risankizumab and TDM assays may differ elsewhere. Authors could expand the discussion on global applicability.	Thank you for your comment. We agree that the findings of this study are likely to be relevant beyond the UK. Although risankizumab has been chosen as the exemplar biologic for psoriasis, the dose minimisation strategies tested in this feasibility trial could, in principle, be applied to other biologics. The increasing availability of biosimilars has improved access to biologic therapies globally, and TDM assays are widely available in laboratories internationally. Once proof-of-principle work has been established for TDM-guided dosing intervals in risankizumab, other drugs could be incorporated into the TDM dashboard, including biosimilars, which may be more accessible in resource-limited settings. We have amended the manuscript to expand the discussion on global applicability.	Lines 623-624

Thank you for reconsidering our submission. We look forward to your response.

VERSION 2 - REVIEW

Reviewer 1

Name Balser, Sigrid

Affiliation Formycon AG, Clinical Development & Operations

Date 25-Sep-2025

COI

Thank you very much for the revision. All my comments have been adequately addressed.
